# 4,5,6,7-Tetra­hydro­benzo[*d*]thia­zol-2-amine

**DOI:** 10.1107/S2414314625009137

**Published:** 2025-10-24

**Authors:** Fatjonë Krasniqi, Gašper Tavčar, Evgeny Goreshnik, Emil Popovski, Ahmed Jashari

**Affiliations:** a Alma Mater Europaea Campus College, ’Rezonanca’, Prishtina 10000, Republic of Kosovo; b Institute of Chemistry, Faculty of Natural Sciences & Mathematics, Ss. Cyril & Methodius University, PO Box 162, Skopje 1000, Republic of North Macedonia; c Department of Inorganic Chemistry and Technology, Jožef Stefan Institute, Ljubljana, 1000, Slovenia; d Group of Chemistry, Faculty of Natural Sciences & Mathematics, University of Tetovo, Tetovo 1200, Republic of North Macedonia; e NanoAlb, Albanian Unit of Nanosciences and Nanotechnology, Academy of Sciences of Albania, Fan Noli square, 1000 Tirana, Albania; University of Aberdeen, United Kingdom

**Keywords:** crystal structure, heterocycles, Hantzsch reaction

## Abstract

In the title compound, C_7_H_10_N_2_S, the six-membered ring is disordered over two half-chair orientations. In the crystal, infinite [001] chains linked by N—H⋯N hydrogen bonds occur.

## Abstract 2

In the title compound, the six-membered ring is disordered over two half-chair orientations. In the crystal, infinite [001] chains linked by N—H⋯N hydrogen bonds occur.

## Graphical abstract

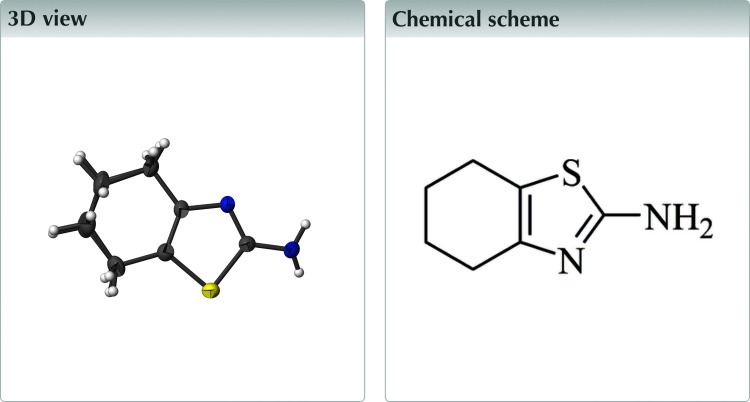

## Structure description

**Figure 1:**
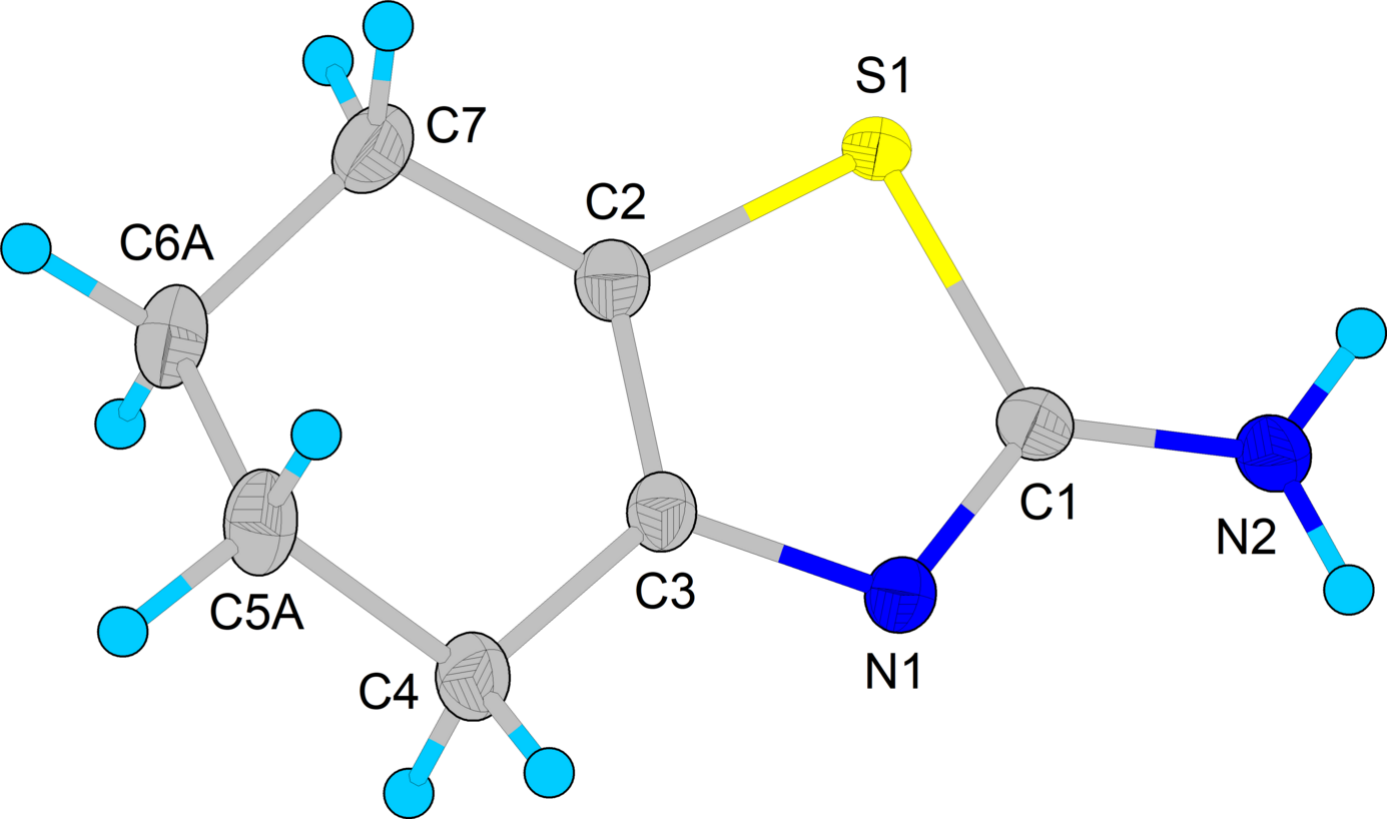
The mol­ecular structure of **I** with displacement ellipsoids drawn at 50% probability. Only the major disorder component for the C5 and C6 methyl­ene groups is shown.

Because of its many pharmacological uses, thia­zole makes an excellent pharmacophore nucleus. Various biological properties such as anti­oxidant (Petrou *et al.*, 2021 ▸) and analgesic (Ye *et al.*, 2013 ▸) activities are exhibited by its derivatives. As part of our studies in this area we now report the synthesis and structure of the title compound, C_7_H_10_N_2_S (Fig. 1 ▸).

As expected, the N atom of thia­zole ring demonstrates noticeable inequality of C—N bond distances [1.391 (2) for C3—N1 *versus* 1.303 (2) Å for C1=N1] corresponding to the presence of single and double bonds. The C2=C3 double bond is clearly shorter [1.344 (3) Å] than the other bonds in six-membered ring. The cyclo­hexene ring is partially disordered (C5 and C6 and their attached H atoms) over two half-chair orientations with very unequal [0.919 (4) *versus* 0.081 (4)] occupancies.

**Figure 2:**
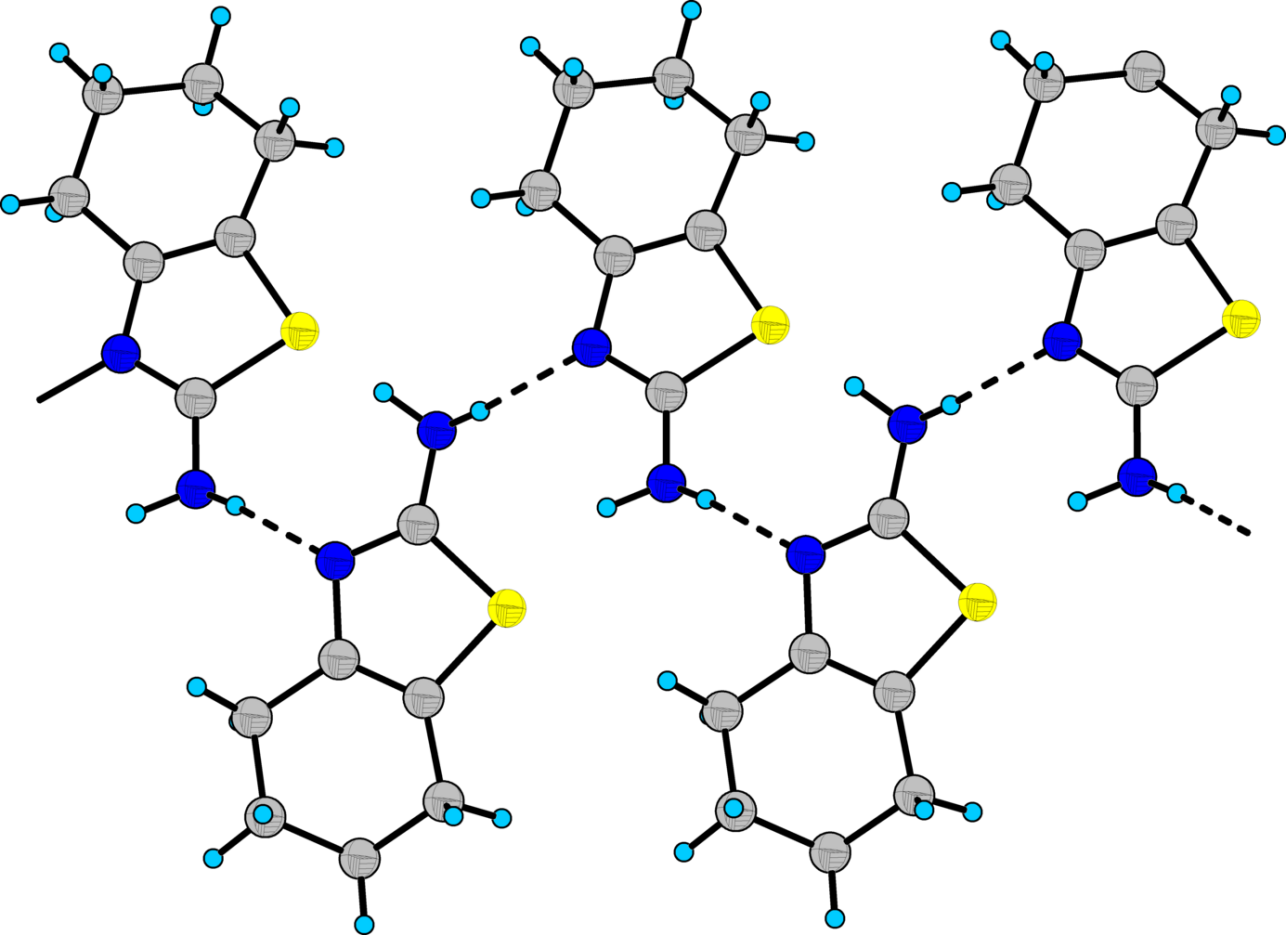
Fragment of a [001] chain of mol­ecules linked by N—H⋯N hydrogen bonds in the crystal structure of **I**.

In the extended structure, the mol­ecules are linked by strong N2—H2*B*⋯N1 hydrogen bonds (Table 1 ▸, Fig. 2 ▸) between the amino group of one mol­ecule and the thia­zole N atom of another mol­ecule to generate infinite chains running along the crystallographic *c-*axis direction. Weaker N2—H2*A*⋯N1 bonds reinforce the chains.

A total of 40 structures containing a 4,5,6,7-tetra­hydro­benzo[*d*]thia­zol-2-amine core were found in a search of the Cambridge Structural Database (CSD, Version 5.45, update of March 2024; Groom *et al.*, 2016 ▸). Excluding compounds with conjugated fragments and protonated salts one may find only five derivatives, namely: 6-nitro-5-phenyl-*N*,*N*-bis­(propan-2-yl)-4,5,6,7-tetra­hydro-1,3-benzo­thia­zol-2-amine (Richter *et al.*, 2018 ▸), *N*-(6-hy­droxy-4,5,6,7-tetra­hydro-1,3-benzo­thia­zol-2-yl)acetamide monohydrate and *N*-(6-hy­droxy-4,5,6,7-tetra­hydro-1,3-benzo­thia­zol-2-yl)acetamide (Ciceri *et al.*, 2020 ▸), 2-amino-4,4,7,7-tetra­methyl-4,5,6,7-tetra­hydro-1,3-benzo­thia­zol-3-ium 4,4,7,7-tetra­methyl-4,5,6,7-tetra­hydro-1,3-benzo­thia­zol-2-amine 3-carb­oxy­propano­ate (Shaibah *et al.*, 2019 ▸) and 3-(4-meth­oxy­phen­yl)-2-(4,5,6,7-tetra­hydro-1,3-benzo­thia­zol-2-yl)prop-2-ene­nitrile (Dyachenko *et al.*, 2021 ▸).

## Synthesis and crystallization

**Figure 3:**
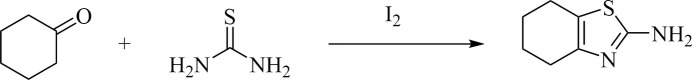
Reaction scheme.

61.13 mmol of thio­urea (4.65 g) and 30.57 mmol (7.76 g) of iodine were mixed in a round-bottom flask. Then, 30.57 mmol (3 g) of cyclo­hexa­none was added and the mixture was refluxed for 24 h at 100 °C. After 24 h, the system was taken out of the oil bath and left to cool to room temperature, meanwhile 350 ml of distilled water was heated to boiling and used in portions to dissolve the reaction mass, and everything was transferred to a crystallizing dish and left to cool to room temperature. Then, three extractions were made with 55 ml of diethyl ether to remove the unreacted ketone, I_2_ and sulfur. Next, 50 ml of NH_4_OH (25%) solution was added to the aqueous solution, and three extractions were made with diethyl ether (55 ml): the ethereal layers were combined, and dried over MgSO_4_. After drying the organic layer was evaporated on a rotavapor. The product was obtained as a light-yellow precipitate. Colourless prisms were recrystallized from *n*-hexane solution. The reaction scheme is shown in Fig. 3 ▸.

FTIR (ATR/cm^−1^): 3430–3250 (NH_2_, stretching), 2850–2950 (CH_2_, asymmetric stretching). 3430 (NH_2_, asymmetric stretching), 3250 (NH_2_, symmetric stretching), 3150, 3100, 3050 (CH, aromatic stretching), 1650 (NH_2_, bending deformations). ^13^C-NMR(δ/p.p.m.: 165.86, 145.35, 114,96, 26.75, 23.72, 23.17, 23.04. ^1^H-NMR (DMSO-*d*_6_, δ/p.p.m.): 6.58 (2*H*, *s*), 2.51 (2*H*, *t*), 2.50 (2*H*, *t*), 2.48 (4*H*, *m*). Elemental analysis: calculated C 54.51; H 6.54; N,18.16. Found: C 54.75; H 7.04; N 18.32. TOF MS ES+(: *m/e*): 155 [*M* + H]^+^, 177 [*M* + Na]^+^.

## Refinement

Crystal data, data collection and structure refinement details are summarized in Table 2 ▸.

## Supplementary Material

Crystal structure: contains datablock(s) I. DOI: 10.1107/S2414314625009137/hb4540sup1.cif

Structure factors: contains datablock(s) I. DOI: 10.1107/S2414314625009137/hb4540Isup2.hkl

Supporting information file. DOI: 10.1107/S2414314625009137/hb4540Isup3.cml

CCDC reference: 2496861

Additional supporting information:  crystallographic information; 3D view; checkCIF report

## Figures and Tables

**Table 1:** Hydrogen-bond geometry (Å, °)

*D*—H⋯*A*	*D*—H	H⋯*A*	*D*⋯*A*	*D*—H⋯*A*
N2—H2*A*⋯N1^i^	0.93 (2)	2.56 (2)	3.392 (2)	149 (2)
N2—H2*B*⋯N1^ii^	0.81 (2)	2.13 (2)	2.941 (2)	175 (2)

Symmetry codes: (i) 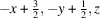; (ii) 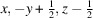.

**Table 2:** Experimental details

Crystal data	
Chemical formula	C_7_H_10_N_2_S
*M*_r_	154.23
Crystal system, space group	Orthorhombic, *Pccn*
Temperature (K)	150
*a*, *b*, *c* (Å)	14.4368 (6), 13.3928 (6), 8.0734 (4)
*V* (Å^3^)	1560.99 (12)
*Z*	8
Radiation type	Cu *K*α
μ (mm^−1^)	3.05
Crystal size (mm)	0.42 × 0.13 × 0.09

Data collection	
Diffractometer	New Gemini, Dual, Cu at home/near, Atlas
Absorption correction	Analytical (*CrysAlis PRO*; Rigaku OD, 2024 ▸)
*T*_min_, *T*_max_	0.484, 0.782
No. of measured, independent and observed [*I* > 2σ(*I*)] reflections	4399, 1606, 1284
*R*_int_	0.037
(sin θ/λ)_max_ (Å^−1^)	0.630

Refinement	
*R*[*F*^2^ > 2σ(*F*^2^)], *wR*(*F*^2^), *S*	0.036, 0.101, 1.00
No. of reflections	1606
No. of parameters	106
H-atom treatment	H atoms treated by a mixture of independent and constrained refinement
Δρ_max_, Δρ_min_ (e Å^−3^)	0.28, −0.23

Computer programs: *CrysAlis PRO* (Rigaku OD, 2024 ▸), *SHELXT2014/4* (Sheldrick, 2015*a* ▸), *SHELXL2016/6* (Sheldrick, 2015*b* ▸) and *OLEX2* (Dolomanov *et al.*, 2009 ▸).

## full crystallographic data

### 4,5,6,7-Tetrahydrobenzo[d]thiazol-2-amine Crystal data

**Table:**

C_7_H_10_N_2_S	*D*_x_ = 1.313 Mg m^−^^3^
*M**_r_* = 154.23	Cu *K*α radiation, λ = 1.54184 Å
Orthorhombic, *P**c**c**n*	Cell parameters from 2022 reflections
*a* = 14.4368 (6) Å	θ = 4.5–75.3°
*b* = 13.3928 (6) Å	µ = 3.05 mm^−^^1^
*c* = 8.0734 (4) Å	*T* = 150 K
*V* = 1560.99 (12) Å^3^	Prism, colourless
*Z* = 8	0.42 × 0.13 × 0.09 mm
*F*(000) = 656	

### 4,5,6,7-Tetrahydrobenzo[d]thiazol-2-amine Data collection

**Table:**

New Gemini, Dual, Cu at home/near, Atlas diffractometer	1606 independent reflections
Radiation source: fine-focus sealed X-ray tube, Enhance (Cu) X-ray Source	1284 reflections with *I* > 2σ(*I*)
Graphite monochromator	*R*_int_ = 0.037
Detector resolution: 10.6426 pixels mm^-1^	θ_max_ = 76.2°, θ_min_ = 4.5°
ω scans	*h* = −17→18
Absorption correction: analytical (CrysAlisPro; Rigaku OD, 2024)	*k* = −14→16
*T*_min_ = 0.484, *T*_max_ = 0.782	*l* = −9→7
4399 measured reflections	

### 4,5,6,7-Tetrahydrobenzo[d]thiazol-2-amine Refinement

**Table:**

Refinement on *F*^2^	Primary atom site location: dual
Least-squares matrix: full	Hydrogen site location: mixed
*R*[*F*^2^ > 2σ(*F*^2^)] = 0.036	H atoms treated by a mixture of independent and constrained refinement
*wR*(*F*^2^) = 0.101	*w* = 1/[σ^2^(*F*_o_^2^) + (0.0586*P*)^2^ + 0.0974*P*] where *P* = (*F*_o_^2^ + 2*F*_c_^2^)/3
*S* = 1.00	(Δ/σ)_max_ = 0.001
1606 reflections	Δρ_max_ = 0.28 e Å^−^^3^
106 parameters	Δρ_min_ = −0.23 e Å^−^^3^
0 restraints	

### 4,5,6,7-Tetrahydrobenzo[d]thiazol-2-amine Special details

**Table:**

Geometry. All esds (except the esd in the dihedral angle between two l.s. planes) are estimated using the full covariance matrix. The cell esds are taken into account individually in the estimation of esds in distances, angles and torsion angles; correlations between esds in cell parameters are only used when they are defined by crystal symmetry. An approximate (isotropic) treatment of cell esds is used for estimating esds involving l.s. planes.
Refinement. 1. Fixed Uiso At 1.2 times of: All C(H,H) groups, All C(H,H,H,H) groups 2. Uiso/Uaniso restraints and constraints Uanis(C5B) = Uanis(C5A) Uanis(C6B) = Uanis(C6A) 3. Others Sof(H4BC)=Sof(H4BD)=Sof(C5B)=Sof(H5BA)=Sof(H5BB)=Sof(C6B)=Sof(H6BA)=Sof(H6BB)= Sof(H7BC)=Sof(H7BD)=1-FVAR(1) Sof(H4AA)=Sof(H4AB)=Sof(C5A)=Sof(H5AA)=Sof(H5AB)=Sof(C6A)=Sof(H6AA)=Sof(H6AB)= Sof(H7AA)=Sof(H7AB)=FVAR(1) 4.a Secondary CH2 refined with riding coordinates: C4(H4AA,H4AB), C4(H4BC,H4BD), C5A(H5AA,H5AB), C5B(H5BA,H5BB), C6A(H6AA,H6AB), C6B(H6BA,H6BB), C7(H7AA,H7AB), C7(H7BC,H7BD)The hydrogen atoms of the amino group were localized in difference Fourier maps and refined freely. Other H-atoms were placed at calculated positions and refined as riding atoms with *U*_iso_(H) = 1.2*U*_eq_(carrier).

### 4,5,6,7-Tetrahydrobenzo[d]thiazol-2-amine Fractional atomic coordinates and isotropic or equivalent isotropic displacement parameters (Å^2^)

**Table:**

	*x*	*y*	*z*	*U*_iso_*/*U*_eq_	Occ. (<1)
S1	0.58597 (3)	0.40079 (3)	0.15755 (6)	0.02956 (17)	
N1	0.65849 (10)	0.36334 (11)	0.44247 (18)	0.0258 (3)	
N2	0.63974 (11)	0.22015 (11)	0.2777 (2)	0.0287 (3)	
H2A	0.6776 (17)	0.1863 (19)	0.352 (3)	0.038 (6)*	
H2B	0.6455 (17)	0.2007 (18)	0.183 (3)	0.035 (6)*	
C1	0.63374 (11)	0.31960 (13)	0.3052 (2)	0.0240 (3)	
C2	0.60279 (12)	0.49990 (13)	0.2947 (2)	0.0268 (4)	
C3	0.64083 (11)	0.46534 (12)	0.4353 (2)	0.0244 (3)	
C4	0.66431 (13)	0.53193 (13)	0.5791 (2)	0.0319 (4)	
H4AA	0.724328	0.511269	0.627540	0.038*	0.919 (4)
H4AB	0.616144	0.525577	0.665786	0.038*	0.919 (4)
H4BC	0.732233	0.540469	0.588221	0.038*	0.081 (4)
H4BD	0.640952	0.502830	0.683839	0.038*	0.081 (4)
C5A	0.67030 (17)	0.64067 (15)	0.5214 (3)	0.0369 (5)	0.919 (4)
H5AA	0.673707	0.685204	0.619040	0.044*	0.919 (4)
H5AB	0.727418	0.650166	0.455371	0.044*	0.919 (4)
C5B	0.615 (2)	0.6383 (18)	0.543 (4)	0.0369 (5)	0.081 (4)
H5BA	0.546954	0.631106	0.557296	0.044*	0.081 (4)
H5BB	0.636943	0.688128	0.624377	0.044*	0.081 (4)
C6A	0.58632 (17)	0.66856 (16)	0.4168 (3)	0.0375 (6)	0.919 (4)
H6AA	0.589342	0.740445	0.388740	0.045*	0.919 (4)
H6AB	0.529217	0.657193	0.482040	0.045*	0.919 (4)
C6B	0.635 (2)	0.6745 (18)	0.373 (4)	0.0375 (6)	0.081 (4)
H6BA	0.701811	0.671058	0.350239	0.045*	0.081 (4)
H6BB	0.613909	0.744546	0.360579	0.045*	0.081 (4)
C7	0.58170 (14)	0.60702 (14)	0.2562 (3)	0.0351 (4)	
H7AA	0.519122	0.612469	0.206832	0.042*	0.919 (4)
H7AB	0.627174	0.633060	0.175189	0.042*	0.919 (4)
H7BC	0.514371	0.619176	0.267818	0.042*	0.081 (4)
H7BD	0.599574	0.622040	0.140437	0.042*	0.081 (4)

### 4,5,6,7-Tetrahydrobenzo[d]thiazol-2-amine Atomic displacement parameters (Å^2^)

**Table:**

	*U*^11^	*U*^22^	*U*^33^	*U*^12^	*U*^13^	*U*^23^
S1	0.0339 (3)	0.0302 (3)	0.0246 (3)	0.00217 (16)	−0.00544 (16)	−0.00116 (16)
N1	0.0307 (7)	0.0205 (7)	0.0261 (7)	−0.0012 (5)	−0.0006 (6)	−0.0002 (5)
N2	0.0369 (8)	0.0238 (7)	0.0254 (8)	−0.0029 (6)	−0.0002 (6)	−0.0022 (6)
C1	0.0226 (7)	0.0263 (8)	0.0231 (8)	−0.0018 (6)	0.0015 (6)	0.0001 (6)
C2	0.0246 (7)	0.0264 (9)	0.0293 (9)	0.0007 (6)	0.0016 (6)	−0.0011 (7)
C3	0.0238 (7)	0.0212 (7)	0.0282 (8)	−0.0008 (6)	0.0031 (6)	−0.0007 (6)
C4	0.0377 (9)	0.0239 (8)	0.0340 (10)	−0.0003 (7)	−0.0045 (7)	−0.0050 (7)
C5A	0.0385 (13)	0.0227 (9)	0.0494 (13)	−0.0015 (8)	−0.0033 (10)	−0.0075 (9)
C5B	0.0385 (13)	0.0227 (9)	0.0494 (13)	−0.0015 (8)	−0.0033 (10)	−0.0075 (9)
C6A	0.0382 (12)	0.0255 (9)	0.0489 (14)	0.0074 (9)	0.0026 (10)	−0.0016 (9)
C6B	0.0382 (12)	0.0255 (9)	0.0489 (14)	0.0074 (9)	0.0026 (10)	−0.0016 (9)
C7	0.0362 (9)	0.0293 (9)	0.0398 (11)	0.0080 (7)	−0.0002 (8)	0.0061 (8)

### 4,5,6,7-Tetrahydrobenzo[d]thiazol-2-amine Geometric parameters (Å, º)

**Table:**

S1—C1	1.7548 (17)	C5A—H5AA	0.9900
S1—C2	1.7453 (18)	C5A—H5AB	0.9900
N1—C1	1.303 (2)	C5A—C6A	1.524 (3)
N1—C3	1.391 (2)	C5B—H5BA	0.9900
N2—H2A	0.93 (3)	C5B—H5BB	0.9900
N2—H2B	0.81 (3)	C5B—C6B	1.48 (4)
N2—C1	1.353 (2)	C6A—H6AA	0.9900
C2—C3	1.344 (3)	C6A—H6AB	0.9900
C2—C7	1.499 (2)	C6A—C7	1.538 (3)
C3—C4	1.503 (2)	C6B—H6BA	0.9900
C4—H4AA	0.9900	C6B—H6BB	0.9900
C4—H4AB	0.9900	C6B—C7	1.51 (3)
C4—H4BC	0.9900	C7—H7AA	0.9900
C4—H4BD	0.9900	C7—H7AB	0.9900
C4—C5A	1.532 (3)	C7—H7BC	0.9900
C4—C5B	1.62 (3)	C7—H7BD	0.9900
			
C2—S1—C1	89.19 (8)	C6A—C5A—H5AB	109.5
C1—N1—C3	110.86 (15)	C4—C5B—H5BA	109.3
H2A—N2—H2B	113 (2)	C4—C5B—H5BB	109.3
C1—N2—H2A	114.4 (16)	H5BA—C5B—H5BB	108.0
C1—N2—H2B	118.6 (18)	C6B—C5B—C4	112 (2)
N1—C1—S1	114.00 (13)	C6B—C5B—H5BA	109.3
N1—C1—N2	124.38 (16)	C6B—C5B—H5BB	109.3
N2—C1—S1	121.56 (14)	C5A—C6A—H6AA	109.3
C3—C2—S1	109.32 (13)	C5A—C6A—H6AB	109.3
C3—C2—C7	126.01 (17)	C5A—C6A—C7	111.73 (18)
C7—C2—S1	124.62 (15)	H6AA—C6A—H6AB	107.9
N1—C3—C4	120.63 (16)	C7—C6A—H6AA	109.3
C2—C3—N1	116.63 (16)	C7—C6A—H6AB	109.3
C2—C3—C4	122.73 (16)	C5B—C6B—H6BA	110.4
C3—C4—H4AA	109.7	C5B—C6B—H6BB	110.4
C3—C4—H4AB	109.7	C5B—C6B—C7	107 (2)
C3—C4—H4BC	110.4	H6BA—C6B—H6BB	108.6
C3—C4—H4BD	110.4	C7—C6B—H6BA	110.4
C3—C4—C5A	109.98 (16)	C7—C6B—H6BB	110.4
C3—C4—C5B	106.4 (10)	C2—C7—C6A	109.22 (17)
H4AA—C4—H4AB	108.2	C2—C7—C6B	109.8 (10)
H4BC—C4—H4BD	108.6	C2—C7—H7AA	109.8
C5A—C4—H4AA	109.7	C2—C7—H7AB	109.8
C5A—C4—H4AB	109.7	C2—C7—H7BC	109.7
C5B—C4—H4BC	110.4	C2—C7—H7BD	109.7
C5B—C4—H4BD	110.4	C6A—C7—H7AA	109.8
C4—C5A—H5AA	109.5	C6A—C7—H7AB	109.8
C4—C5A—H5AB	109.5	C6B—C7—H7BC	109.7
H5AA—C5A—H5AB	108.0	C6B—C7—H7BD	109.7
C6A—C5A—C4	110.91 (18)	H7AA—C7—H7AB	108.3
C6A—C5A—H5AA	109.5	H7BC—C7—H7BD	108.2

### 4,5,6,7-Tetrahydrobenzo[d]thiazol-2-amine Hydrogen-bond geometry (Å, º)

**Table:**

*D*—H···*A*	*D*—H	H···*A*	*D*···*A*	*D*—H···*A*
N2—H2*A*···N1^i^	0.93 (2)	2.56 (2)	3.392 (2)	149 (2)
N2—H2*B*···N1^ii^	0.81 (2)	2.13 (2)	2.941 (2)	175 (2)

Symmetry codes: (i) −*x*+3/2, −*y*+1/2, *z*; (ii) *x*, −*y*+1/2, *z*−1/2.
